# Language and Measurement of Contraceptive Need and Making These Indicators More Meaningful for Measuring Fertility Intentions of Women and Girls

**DOI:** 10.9745/GHSP-D-21-00450

**Published:** 2022-02-28

**Authors:** Ilene S. Speizer, Jason Bremner, Shiza Farid

**Affiliations:** a Department of Maternal and Child Health and Carolina Population Center, University of North Carolina Gillings School of Global Public Health, Chapel Hill, NC, USA.; b FP2030, Washington DC, USA.; c Avenir Health, Washington, DC, USA.

## Abstract

We examine current “need”-based family planning measures that are based on women’s fertility desires and contraceptive use, identify challenges with language and use of need-based measures, and recommend ways to improve language and measurement.

## Key Messages

- “Need”-based measures, such as unmet need and demand satisfied, are indicators used at the global level to assess progress in the family planning (FP) field and provide strong justification for FP programs.
- These measures are commonly misused or misinterpreted and might not actually represent what girls and women want or need in terms of contraceptive methods and services.
- To strengthen understanding and use, we recommend that inaccurate and confusing language be removed from the names and labels of these measures and that the FP field work to identify more accurate language.
- Using a human rights and reproductive justice lens, the field needs to do 2 things: (1) consider refinements to current measures that better capture self-identified needs and preferences; and (2) develop new measures that capture the perspectives of users, potential users, and non-users; this likely requires formative research with women, men, and couples on motivations, aspirations, and preferences around fertility desires and contraceptive use.

FP2030 (https://www.FP2030.org) is the next phase of the FP2020 global partnership which has the vision of a future where all women and girls, no matter where they live, have the freedom and ability to make their own informed decisions about using modern contraception and whether or when to have children. FP2030 recognizes the importance of women and girls’ autonomy in seeking and using family planning (FP) services if and when they choose. As part of this initiative, the Performance, Monitoring and Evidence Working Group (PME-WG), an independent group of FP measurement experts from a wide range of institutions, will support the monitoring of policy and program efforts and accountability through routine review of country-level core indicators (https://www.fp2030.org/get-data). As the PME-WG establishes the measures to monitor FP2030, the group recognizes the need to review and expand its measurement framework and indicators to align them with the increased women-centered focus of FP2030.

As part of this effort, we feel it is time to highlight problematic language and misuse of common FP measures, particularly **unmet need** and **demand satisfied**, terms that fall short of capturing women’s (and their partners’) preferences and intentions around fertility and contraceptive use.

In low- and middle-income countries, 218 million women are estimated to have an unmet need for modern contraception, meaning they do not want to have a child in the next 2 years or at all and are not using a modern method, are using a traditional method, or are pregnant with or postpartum amenorrheic after an unintended pregnancy.^1^

This commentary employs a human rights and reproductive justice lens that focuses on ensuring that all people have the agency and autonomy to freely decide whether and when to have children and whether or not to use contraception and that they have access to high-quality information and services offering a range of methods in a nondiscriminatory manner.^2^^–^^4^ Using this lens, we examine current “need”-based FP measures, that is, measures that are based on women’s fertility desires and contraceptive use, by (1) summarizing the history and definitions of these measures^*^; (2) identifying challenges with language and use of need-based measures; and (3) proposing ways forward for improving language and measurement.

## HISTORICAL PERSPECTIVE OF NEED-BASED MEASURES

Unmet need was originally referred to as the knowledge, attitudes, and practices (KAP) gap, or “KAP-gap,” taking its name from a suite of surveys fielded in the 1960s developed to capture KAPs of women to permit direct examination of birth rates, fertility desires, and contraceptive use behaviors. KAP surveys were followed by World Fertility Surveys (WFS) and then Demographic and Health Surveys (DHS).^†^Across these country-based surveys, significant proportions of women married or living with a partner in union reported they desired to limit fertility and were not using contraception; this was referred to as the “KAP-gap.”^5^ The KAP-gap was later renamed “unmet need for family planning” by Westoff in analyses of WFS data from Asia.^6^^,^^7^

The Programme of Action from the 1994 International Conference on Population and Development (ICPD) emphasized meeting the fertility desires of individual women and men, instead of achieving demographic targets.^8^ This move toward meeting women’s and couples’ reproductive intentions aligned well with the concept of “unmet need for family planning”—the percentage of women who have the intention to avoid pregnancy but are not using contraception to meet that intention.

The measurement of unmet need has been refined over time using DHS data to better measure the pool of women who are not using contraception who represent the potential demand for contraception, called “total unmet need.” The revised measure was meant to address identified problems with the KAP-gap by including spacing preferences and acknowledging that some women were pregnant or postpartum amenorrhoeic at the time of the survey resulting from a pregnancy that was mistimed or unwanted and that they likely had unmet need before they became pregnant.^9^ Additional refinements improved measurement of fecundability and included sexually active unmarried women (Box 1).^6^^,^^10^^,^^11^

BOX 1 **Definitions of Unmet Need and Demand for Contraception**

**Unmet need:** The definition of unmet need now used by the Demographic and Health Survey, United Nation agencies, and donors relies on an algorithm based on 15 survey questions^11^ and considers women to have unmet need if they are not currently using contraception; are either

(1) currently married or living with a partner in union, or

(2) unmarried and had sex in the past 30 days; and:

- Are fecund and do not want children in the next 2 years (unmet need for spacing)
- Are fecund and do not want any (more) children (unmet need for limiting)
- Are pregnant or postpartum amenorrheic after a mistimed pregnancy (unmet need for spacing), or
- Are pregnant or postpartum amenorrheic after an unwanted pregnancy (unmet need for limiting)

**Total demand for contraception** is defined as the percentage of women who are using contraception (contraceptive prevalence [CP]) plus the percentage of women with an unmet need.

**Demand satisfied** is the percent of total demand that is satisfied with contraception, i.e., CP/(unmet need + CP).

**Demand satisfied for modern methods** is the percentage of women using modern methods (MCP) divided by the total demand for modern methods, i.e., MCP/(unmet need for modern + MCP).

Women who are infecund or who are unmarried and have not had sex in the last 30 days are considered to have no need for contraception.^12^ A major criticism of the measure has been that the unmet need definition does not include questions about whether women want or intend to use contraception.^13^

A major criticism of the measure has been that the unmet need definition does not include questions about whether women want or intend to use contraception.

Over time, unmet need went from solely a demographic measure to a key indicator used to measure progress across global calls to action, including Millennium Development Goal 5b. The indicator later became key to UNFPA’s ICPD+25 transformational goal of zero unmet need and part of the FP2020 and now FP2030 indicators.

Additional indicators that relate to **unmet need** have been developed and are now part of global measurement agendas including **total demand for contraception** and **demand satisfied** (Box 1). **Demand satisfied** assesses the overall proportion of women who want to delay or avoid childbearing who are currently using contraception themselves or are relying on their partners’ method use.^14^ This measure is considered by some to reflect voluntarism and informed choice because it does not set contraceptive prevalence nor fertility targets but rather emphasizes the imperative to satisfy individuals’ and couples’ own choices regarding the number and timing of children.^15^ That said, **demand satisfied**, like unmet need, does not take into consideration whether women are freely choosing to use a method nor if they are using the method of their choice.^16^ Also, like unmet need, it assumes that all married women are exposed to the risk of pregnancy, which is demonstrably untrue and biases measures of need upward.^17^ **Demand satisfied with modern methods** is a key indicator for Sustainable Development Goal target 3.7 to ensure universal access to sexual and reproductive health care services by 2030.

## CHALLENGES WITH LANGUAGE AND USE OF NEED-BASED MEASURES

While the measurement of unmet need, demand satisfied, and total demand has evolved and improved, the use of these measures has consistently been a challenge. Unmet need and demand satisfied have frequently been misinterpreted as reflections of women’s intentions—that women who are classified as having unmet need or demand want to use contraception. While this would appear to be a logical interpretation based on the terms "need” and “demand," unmet need does not actually measure a woman's individual preferences for contraception, only the gap between reported fertility preferences and current contraceptive use. In fact, while proportions vary widely by country, overall about half of the women who are classified as having unmet need based on their responses to survey questions about their fertility intentions and contraceptive use say in that same interview that they do not want nor intend to use contraception in the future.^18^ Further, many women have ambivalent fertility desires (i.e., are undecided about future childbearing or the timing of future childbearing).^19^ Such women may be misclassified as having an unmet need when indeed they have no desire or intention to use a method in the future and may welcome an “unexpected” pregnancy,^20^^–^^22^ although the majority of women with unintended pregnancies obtain abortions.^23^

As noted previously, problems with language that is currently used to describe need-related indicators are further illustrated through the label of “demand satisfied” for contraception and especially “demand satisfied for modern methods.” An individual’s preference may not be satisfied in the case where a woman may have limited method choices or where she is dissatisfied with the use requirements or side effects of her current method.^24^^,^^25^ Further, demand typically reflects what a woman (or couple) wants and is willing to do. Thus, to assess demand satisfied, we would want to ask people more directly what they want, whether they have the agency/capability to realize this option, and what satisfaction means to them.^26^

To assess demand satisfied, we would want to ask people more directly what they want, whether they have the agency/capability to realize this option, and what satisfaction means to them.

At an individual level, the seeming discrepancy between intentions and use can be understood as a woman’s personal cost-benefit analysis: she may want to avoid pregnancy, but that “benefit” of avoiding pregnancy may not be large or strong enough to outweigh the “costs” associated with contraceptive use or with use of specific methods. These “costs” may include those related to supply such as financial and/or time costs to access and obtain the method of her choice as well as those related to personal or social issues, such as experienced or feared side effects, potential partner or community opposition of contraception, and health concerns, among others.^27^^,^^28^ From a reproductive justice perspective, based on established human rights,^29^^,^^30^ the individual has the right to choose to use or not to use a contraceptive method to meet her fertility desires, and thus, her self-reported needs and wants must be assessed to better understand gaps in use, recognizing that some women may also be ambivalent toward pregnancy and/or contraceptive use.

Some girls and women, as well as people who do not identify as female, also use contraception for nonfertility-related reasons including menstrual control, acne prevention, or prevention of sexually transmitted infections, or may use methods for other noncontraceptive benefits. These people would be considered to have no unmet need for contraception based on their fertility intentions; however, as with other groups of users, they may not be using the method of their choice or may not be satisfied with their contraception.

As pointed out in their article that examines the history, use, and measurement of unmet need, Bradley and Casterline^6^ clarify that unmet need was never meant to indicate that the full population of women with an unmet need (e.g., 218 million women with an unmet need for modern methods in low- and middle-income countries as listed previously) lack access and would use a modern method if FP programs were made available. Unmet need and demand satisfied measures were meant to indicate what levels of contraceptive use and subsequent fertility rates would be achieved at the population level in a scenario where FP is universally available and accessible and all women wanting to avoid pregnancy chose to use contraception.

For program and policy makers, measures of unmet need have been used to identify potential gaps in service delivery and inequities in services. However, unmet need indicators do not solely or specifically reflect inadequacies in service delivery.^13^ Other measures are necessary to assess the local and personal contexts that affect women’s (and their partners’) interest and ability to use contraception.^6^^,^^31^^–^^33^ In particular, unmet need measures fail to reflect whether women know about FP, whether they approve of it, if they intend to use it, and their ability to use it should they want to, that is, they fail to capture the numerous demand and supply-side factors that affect contraceptive use (Box 2).^34^^,^^35^

BOX 2 **Demand- and Supply-Side Barriers to Contraceptive Use**

Some demand-side barriers:

- Experience/fear of side effects
- Do not like available methods
- Partner/self/other opposition
- Gender norms that affect agency and decision making

Some supply-side barriers:

- Stock-outs; limited availability of services and supplies
- Provider bias and poor-quality counseling
- Costs of services and supplies

Further, need-based measures are often used with reference specifically to modern contraceptive methods (e.g., the Sustainable Development Goal indicator of demand satisfied with modern methods and the FP2030 country indicators of unmet need for modern methods and demand satisfied with modern methods). Modern-method-focused indicators consider users of traditional methods to have an “unmet need” or not have their “demand satisfied.” It is important to make clear the implications of defining unmet need for modern methods versus unmet need for any method. A woman or couple’s use of traditional methods may reflect their choice and what is the most appropriate method for their current life phase and situation or it may reflect barriers to their use of a preferred modern method. If we examine these measures with a reproductive justice and human rights perspective,^3^ classifying women using traditional methods (and even nonusers) as “in-need” misses the consideration of whether traditional method use and nonuse is the person’s (couple’s) choice, a reflection of their agency or empowerment to choose a traditional method (or lack of agency or empowerment to choose a different method), or an indicator of difficulty being able to use their desired method. No matter a person’s fertility desires, they still have the right to choose not to use a method or to use a traditional method if that meets their self-identified preferences. Indeed, at the service level, understanding each individual’s desires and preferences and their life contexts is necessary to guide FP outreach and services to meet clients’ actual needs.

No matter a person’s fertility desires, they still have the right to choose not to use a method or to use a traditional method if that meets their self-identified preferences.

Measures of unmet need have also been used to illustrate funding requirements and potential impacts if all women (and their partners) who want to avoid pregnancy use a contraceptive method.^1^^,^^36^ The estimates that result from this type of projection may overestimate service need since, as discussed previously, many women who are classified as having an unmet need may not want to use a method even if it is made freely accessible and available. They also overestimate service needs by assuming all women who are married or who had sex in the past 30 days are at risk of pregnancy if they do not report current contraceptive use.^17^ These estimates may also underestimate service need since some women and couples may be dissatisfied with their current method and if alternatives were offered, they might visit a facility to switch methods.^25^ Given the possibility for over- or underestimation of unmet need and that demand satisfied fails to capture individuals’ actual preferences, these measures need to be reconsidered as the sole global indicators to measure progress toward universal access to FP and sexual and reproductive health services.

## PROPOSED WAYS FORWARD

To look critically at our field and consider our approach to language and measurement in FP, we propose to consider measurement with a new lens. From a human rights and reproductive justice perspective, all people should have complete access to a full range of methods (operationally defined by the World Health Organization as at least 1 short-term, 1 long-term, 1 permanent, and 1 emergency method^4^) to use if or when they choose. However, even this definition may not meet everyone’s needs, particularly if they have preferences for specific short-term (i.e., pill over injection) or long-term (i.e., intrauterine device versus implant) methods; if they need or prefer to avoid hormonal methods; or if they need contraceptives that also prevent against sexually transmitted infections, for example. If geographic/physical, economic, administrative, cognitive, and psychosocial access^32^ are not maintained, gaps in use should not be interpreted as simply a mismatch between intentions and actions. The Sustainable Development Goals seek to advance universal access to sexual and reproductive health. If we were to achieve this goal, we could expect that all people who want to use contraception would have access to and the freedom to choose a method that meets their self-identified preferences—including a traditional method—as well as the freedom to choose not to use a method.

As we examine the next 10 years of global FP efforts under FP2030, now is the time to reconsider the measures and language we use to identify and support people to avoid unintended pregnancy in ways they prefer (Box 3). Notably, it is not that the indicators of unmet need, demand satisfied, and total demand must go away, but rather we can do 2 things: (1) change the language around these indicators to make their meaning clearer and more useful and potentially further refine their measurement; and (2) identify new, more salient indicators from the user perspective to pilot, validate, and hopefully use as we move global FP monitoring efforts forward.

BOX 3 **Recommendations for Refining Family Planning Indicators**

- Improve the language used to describe existing indicators
- Develop new metrics using a human rights and reproductive justice lens
- Engage a broader set of stakeholders, including contraceptive users, in the design of person-centered measures
- Determine if measures need to be different for varying stages of the life course
- Test and validate proposed measures of satisfaction and autonomy to make recommendations for broader use
- Once measures are identified (and validated), develop and disseminate fact sheets and other materials to support standardized language and use

### Changing Language

The meaning and usefulness of the suite of need-based indicators have often been misconstrued, in part because their labeling is often confusing. Now is an opportunity to reconsider these terms, with a reproductive justice/rights perspective. The language around these measures should reflect that some people who do not want to become pregnant are choosing **not** to use contraception and therefore do not have a “need.” In addition, some people choose to use a traditional method and thus do not have a “need” for modern methods, and some who are using a modern method may not be “satisfied” or supported in their use.

For the unmet need indicator, some have proposed an alternative label of “potential demand,”^9^ and the PME-WG has discussed the possibility of using terminology such as “utilization gap.” A fuller, accurate title would be “percent of women who want to avoid pregnancy but are not using contraception.” All of these terms indicate a difference between women’s stated desires to avoid pregnancy and their use of (modern) contraception. The latter 2 labels also avoid implying that women “need” or “demand” contraception, which is of particular importance given that half of the women classified with unmet need do not want nor intend to use contraception.^18^ The term also leaves room for women to have a choice not to use.

For the demand satisfied indicator, a more accurate title would be “percent of women using contraception among women who say they do not want to get pregnant” (or percent using among those who intend to avoid pregnancy) avoiding implying that the woman (or couple) is “satisfied” with the method. Further, total demand might be better labeled “total potential use” for FP.

The PME-WG recognizes that these indicators will remain prominent but intends to engage in and support a lively discussion on how to relabel them and to strengthen the understanding of what they do and do not measure so that they can be accurately interpreted by all. We recognize that renaming these indicators is a first step; however, it is also important to develop factsheets and briefs that reach a broad audience of program planners, policy makers, funders, and researchers to clarify the meaning and interpretation of these indicators.

### Alternative Metrics

Improving the language and understanding of current measures is a first step toward being more realistic about what we are capturing; however, alternative measures also need to be considered. In recent commentaries, Rominski and Stephenson^24^ and Rothschild, Brown, and Drake^25^ propose incorporating women’s satisfaction with their current method into unmet need measurements to better capture women who are underserved by FP programming; this would likely increase the level of need assessed. Alternatively, Moreau et al.^18^ propose a measure called “current status unmet demand” that is measured based on a revised unmet need and intention to use contraception, identifying women interested and willing to use a method; this lowers the level of need. These are good starting points. The Moreau approach can be explored using existing data from the DHS or Performance Monitoring and Accountability data sources; however, satisfaction measures lack standardized, valid approaches to measurement and thus need further refinement for broader assessment. Moving forward, to improve the language and use of these measures, we need to reexamine underlying theory and conceptualizations and reach out to women, men, and couples to pointedly ask them what they want and need in FP programming; what personal and structural barriers stand in the way; and how they make decisions about the cost and benefit of contraceptive use. This is consistent with a new measure proposed by Senderowicz^16^ to address contraceptive autonomy that captures people’s informed choice, full choice, and free will to use (or not) a method. These types of new measures need to be tested to help the FP community understand the nuances of the intention-uptake gap and can be used to design new indicators for use by policy makers and program planners.

From the outset of FP2020 and as we continue into the FP2030 initiative, measurement activities have been built on the recognition that multiple dimensions and factors underlie contraceptive choice and use and that progress under the FP2020 and now the FP2030 initiative should be measured across a range of indicators reflecting rights and empowerment principles, including quality of care, agency and autonomy, informed choice, equity, and transparency and accountability, among others highlighted in FP2020’s Rights and Empowerment Principles (https://commitments.fp2030.org/principles). Thus FP2020/FP2030 measurement efforts and the measurement framework comprise not just 1 indicator but a broader set that speaks to where and how progress is needed and being made. Recently, attention has turned to identifying person-centered population-based indicators for FP to capture women’s and girls’ perspectives on sexual and reproductive health intentions and service use.^16^^,^^37^ Person-centered measures of FP program success have been identified as a gap in our measurement frameworks and are considered part of the unfinished agenda.^38^

Both existing and new measures must be examined, and perhaps reframed, to ensure that perspectives of users, potential users, and nonusers are included. Without this effort, we risk programs and policies emphasizing measurable (i.e., status quo) outcomes such as unmet need and demand satisfied and never moving into the realm of what else should be measured to better direct efforts that meet women’s and girls’ (men’s or couples’) self-defined contraceptive needs. To support this, we need formative research on motivations, aspirations, autonomy, agency, informed decision making, and preferences around fertility desires and contraceptive use that directly obtains this from women, men, and couples. Further, we need to examine whether and how desires, intentions, and use of contraception are anchored to key life events or social milestones and determine if this requires differing measures over the life course. We as a field need to do better to listen to the people being served, understand their self-identified needs, and then develop standardized measures to capture those perspectives and avoid mislabeling and misusing them in the process.

This commentary is a call to action from the PME-WG to the broader FP community (e.g., survey researchers, governments, donors, policy advocates, and program managers) to work toward (1) developing better metrics to improve analysis, policy, and program planning at the country level and (2) testing novel metrics and sharing findings widely. Refining unmet need and related indicators with new language and measures will take time and should involve consultation with a broad range of stakeholders across the social justice and FP communities, and, most importantly, women themselves. We are committed to advancing this process.

## Footnotes

* This commentary does not comprehensively review the field’s complicated history in identifying and measuring contraceptive needs and population policies which was not always rooted in a rights-based, person-centered lens. A detailed history of the measurement of unmet need can be found elsewhere.^6^

## Footnotes

† WFS were nationally representative surveys that were established in 1972 and covered reproductive health household characteristics such as family composition, marital status, economic status, etc. In 1984, the WFS was replaced with the DHS which is also nationally representative and covers a breadth of health and population issues.

## Notes

**Peer Reviewed**

**First published online:** February 24, 2022.

## References

[1] Sully E Biddlecom A Darroch JE. *Adding It Up: Investing in Sexual and Reproductive Health 2019*. Guttmacher Institute; 2020. Accessed May 14, 2021. https://www.guttmacher.org/sites/default/files/report_pdf/adding-it-up-investing-in-sexual-reproductive-health-2019.pdf

[2] FP2030; UNFPA; What Works Association. The Comprehensive Human Rights-based Voluntary Family Planning Program Framework: Brief. FP2030; 2021. Accessed November 12, 2021. https://commitments.fp2030.org/sites/default/files/06.25.21_Framework_Brief.pdf

[3] Ross LJ. Reproductive justice as an intersectional feminist activism. Souls. 2017;19(3):286–314. 10.1080/10999949.2017.1389634

[4] World Health Organization (WHO). *Ensuring Human Rights Within Contraceptive Programs: A Human Rights Analysis of Existing Quantitative Indicators*. WHO; 2014. Accessed March 13, 2021. https://apps.who.int/iris/bitstream/handle/10665/126799/9789241507493_eng.pdf

[5] Westoff CF. Is the KAP-Gap real? Popul Dev Rev. 1988a;14(2):225–232. 10.2307/1973570

[6] Bradley SEK Casterline JB. Understanding unmet need: history, theory, and measurement. Stud Fam Plann. 2014;45(2):123–150. 10.1111/j.1728-4465.2014.00381.x. 24931072 PMC4369378

[7] Westoff CF. The unmet need for birth control in five Asian countries. Fam Plann Perspect. 1978;10(3):173–181. 10.2307/2134309. 658326

[8] United Nations Population Fund (UNFPA). *Programme of Action. Adopted at the International Conference on Population and Development, Cairo, 5–13 September 1994*. UNFPA; 2004. Accessed January 31, 2022. https://www.unfpa.org/sites/default/files/event-pdf/PoA_en.pdf

[9] Westoff CF. The potential demand for family planning: a new measure of unmet need and estimates for five Latin American countries. Int Fam Plan Perspect. 1988b;14(2):45–53. 10.2307/2947679. 12281824

[10] Westoff CF. *Unmet Need at the End of the Century*. DHS Comparative Reports No. 1. ORC Macro; 2001. Accessed February 1, 2022. https://dhsprogram.com/pubs/pdf/CR1/C1.pdf

[11] Bradley SEK Croft TN Fishel JD Westoff C. Revising Unmet Need for Family Planning. DHS Analytical Studies No. 25. ICF International; 2012. Accessed January 31, 2022. https://dhsprogram.com/publications/publication-as25-analytical-studies.cfm

[12] Fabic MS Jadhav A. Standardizing measurement of contraceptive use among unmarried women. Glob Health Sci Pract. 2019;7(4):564–574. 10.9745/GHSP-D-19-00298. 31874938 PMC6927838

[13] Pritchett LH. Desired fertility and the impact of population policies. Popul Dev Rev. 1994;20(1):1–55. 10.2307/2137629

[14] Singh S Darroch JE Vlassoff M Nadeau J. *Adding It Up: The Benefits of Investing in Sexual and Reproductive Health Care*. The Alan Guttmacher Institute, UNFPA; 2003. Accessed January 31, 2022. https://www.guttmacher.org/sites/default/files/report_pdf/addingitup2003.pdf

[15] Fabic MS Choi Y Bongaarts J. Meeting demand for family planning within a generation: the post-2015 agenda. Lancet. 2015;385(9981):1928–1931. 10.1016/S0140-6736(14)61055-2. 24993915 PMC4393371

[16] Senderowicz L. Contraceptive autonomy: conceptions and measurement of a novel family planning indicator. Stud Fam Plann. 2020;51(2):161–176. 10.1111/sifp.12114. 32358789

[17] Bell SO Bishai D. Unmet need and sex: investigating the role of coital frequency in fertility control. Stud Fam Plann. 2017;48(1):39–53. 10.1111/sifp.12012. 28145582

[18] Moreau C Shankar M Helleringer S Becker S. Measuring unmet need for contraception as a point prevalence. BMJ Glob Health. 2019;4(4):e001581–e001581. 10.1136/bmjgh-2019-001581. 31543991 PMC6730575

[19] Withers MH Tavrow P Adinata NA. Do ambivalent women have an unmet need for family planning? A longitudinal study from Bali, Indonesia. Womens Health Issues. 2011;21(6):444–449. 10.1016/j.whi.2011.04.031. 21723743

[20] Trussell J Vaughan B Stanford J. Are all contraceptive failures unintended pregnancies? Evidence from the 1995 National Survey of Family Growth. Fam Plann Perspect. 1999;31(5):246–247, 260. 10.2307/2991573. 10723650

[21] Speizer IS Calhoun LM Hoke T Sengupta R. Measurement of unmet need for family planning: longitudinal analysis of the impact of fertility desires on subsequent childbearing behaviors among urban women from Uttar Pradesh, India. Contraception. 2013;88(4):553–560. 10.1016/j.contraception.2013.04.006. 23706906 PMC3835184

[22] Speizer IS Escamilla V Lance PM Guilkey DK. Longitudinal examination of changing fertility intentions and behaviors over a four-year period in urban Senegal. Reprod Health. 2020;17(1):38. 10.1186/s12978-020-0893-4. 32183890 PMC7077111

[23] Bearak J Popinchalk A Ganatra B. Unintended pregnancy and abortion by income, region, and the legal status of abortion: estimates from a comprehensive model for 1990–2019. Lancet Glob Health. 2020;8(9):e1152–e1161. 10.1016/S2214-109X(20)30315-6. 32710833

[24] Rominski SD Stephenson R. Toward a new definition of unmet need for contraception. Stud Fam Plann. 2019;50(2):195–198. 10.1111/sifp.12084. 30741426

[25] Rothschild CW Brown W Drake AL. Incorporating method dissatisfaction into unmet need for contraception: implications for measurement and impact. Stud Fam Plann. 2021;52(1):95–102. 10.1111/sifp.12146. 33595116 PMC8048066

[26] Bhan N Raj A. From choice to agency in family planning services. Lancet. 2021;398(10295):99–101. 10.1016/S0140-6736(21)00990-9. 33971154

[27] Pitorak H Lubaale SK Gurman TA. “It depends on your pocket:” findings from a qualitative study in Uganda exploring women’s and health care providers’ perspectives on family planning. Health Care Women Int. 2014;35(3):234–248. 10.1080/07399332.2012.736575. 23530463

[28] Farmer DB Berman L Ryan G. Motivations and constraints to family planning: a qualitative study in Rwanda’s southern Kayonza district. Glob Health Sci Pract. 2015;3(2):242–254. 10.9745/GHSP-D-14-00198. 26085021 PMC4476862

[29] United Nations (UN). *Final Act of the International Conference on Human Rights. Teheran. 22 April–13 May, 1968*. UN; 1968.

[30] Erdman JN Cook RJ. Reproductive rights. In: Heggenhougen H.K., ed. *International Encyclopedia of Public Health*. Academic Press; 2008:532–538.

[31] Institute of Medicine. *Contraceptive Research and Development: Looking to the Future*. Harrison PF Rosenfield A, eds. National Academies Press; 1996. 25121263

[32] Bertrand JT Hardee K Magnani RJ Angle MA. Access, quality of care and barriers in family planning programs. Int Fam Plan Perspect. 1995;21(2):64–69, 74. 10.2307/2133525

[33] Potter JE Stevenson AJ Coleman-Minahan K. Challenging unintended pregnancy as an indicator of reproductive autonomy. Contraception. 2019;100(1):1–4. 10.1016/j.contraception.2019.02.005. 30851238 PMC6919552

[34] Sedgh G Hussain R. Reasons for contraceptive nonuse among women having unmet need for contraception in developing countries. Stud Fam Plann. 2014;45(2):151–169. 10.1111/j.1728-4465.2014.00382.x. 24931073

[35] Solo J Festin M. Provider bias in family planning services: a review of its meaning and manifestations. Glob Health Sci Pract. 2019;7(3):371–385. 10.9745/GHSP-D-19-00130. 31515240 PMC6816811

[36] UNFPA Technical Division. *Revised Cost Estimates for the Implementation of the Programme of Action for the International Conference on Population and Development: A Methodological Report*. UNFPA; 2009. Accessed January 31, 2022. https://www.unfpa.org/sites/default/files/resource-pdf/Revised_Costing_ICPD.pdf

[37] Sudhinaraset M Afulani PA Diamond-Smith N Golub G Srivastava A. Development of a person-centered family planning scale in India and Kenya. Stud Fam Plann. 2018;49(3):237–258. 10.1111/sifp.12069. 30069983

[38] Brown W Druce N Bunting J. Developing the “120 by 20” goal for the global FP2020 initiative. Stud Fam Plann. 2014;45(1):73–84. 10.1111/j.1728-4465.2014.00377.x. 24615576

